# Palliative radiotherapy for gastric cancer: Is there a dose relationship between bleeding response and radiotherapy?

**DOI:** 10.6061/clinics/2020/e1644

**Published:** 2020-08-28

**Authors:** Gustavo Arruda Viani, Caio Viani Arruda, Ana Carolina Hamamura, Alexandre Ciufi Faustino, Anielle Freitas Bendo Danelichen, Fernando Kojo Matsuura, Leonardo Vicente Fay Neves

**Affiliations:** I Faculdade de Medicina de Ribeirao Preto (FMRP), Universidade de Sao Paulo, Ribeirao Preto, SP, BR.; II Instituto de Biociencias, Universidade Estadual Paulista (UNESP), Botucatu, SP, BR.

**Keywords:** Palliative, Radiotherapy, Gastric Cancer, Bleeding

## Abstract

The aim of this study was to evaluate whether there is a relationship between bleeding response and radiotherapy dose to palliate patients with local recurrence or progression of gastric cancer (GC). To this end, we conducted a systematic review and meta-analysis of observational studies that evaluated the bleeding response in patients with GC with local recurrence or progression. A meta-regression analysis between biological effective dose (BED) and bleeding response was performed, as was subgroup analysis to evaluate the outcome by BED level and radiotherapy (RT) technique. A *p*-value <0.05 was considered significant.

Ten non-comparative retrospective studies and one prospective study were included. In general, RT was effective at controlling tumor bleeding, and the bleeding response rate was 0.77 (95% confidence interval (CI), 0.73-0.81). Meta-regression analysis demonstrated a linear correlation between BED Gy 10 and bleeding response (*p*=0<0001). Studies using conformational RT had a significant bleeding response rate compared to those using 2D (0.79; 95%CI, 0.74-0.84 *vs* 0.65; 95%CI, 0.56-0.75; *p*=0.021). In terms of the BED level, a significant difference in BR was identified on comparing BED Gy10 ≥40 (0.79; 95%CI, 0.7-0.8), BED Gy10 30-39 (0.79, 95%CI, 0.71-0.86), and BED Gy10 <30 (0.64; 95%CI, 0.5-0.7; *p*=0.0001). The mean survival time was 3.31 months (95%CI, 2.73-3.9) months, and the responders had a significantly longer survival (longer by 2.5 months) compared to the non-responders (95%CI, 1.7-3.3; *p*<0.0001).

Palliative RT is effective at controlling bleeding due to local recurrence/progression from GC. Our findings reveal a relationship between BR and BED. BED <30 Gy 10 should not be recommended, and 3DRT should be indicated instead in order to improve the result.

## INTRODUCTION

Gastric cancer (GC) is one of the most prevalent malignant diseases worldwide, and it results in a significant proportion of cancer-related death (1). Surgical resection is considered the cornerstone of GC treatment and the only treatment with the capacity to lead to long-term survival (2).

However, even with total gastrectomy and extending lymphadenectomy, the rate of local recurrence is high, and adjuvant chemotherapy or chemoradiation is administered with the aim of reducing recurrence (2-5). Local recurrence or local progression from an unresectable disease is a challenging clinical situation for several reasons. First, the majority of patients with local recurrence/progression experience pain, bleeding, and gastric outlet obstruction, which results in a reduced quality of life and a diminished clinical performance (6). Second, local failure of GC is difficult to salvage with chemotherapy and other treatments, and consequently, the prognosis of these patients is poor (6-8). Third, approximately 50% of patients with local recurrence have metastatic disease at the same time (9). In this clinical scenario, the oncologist has several treatment options, including palliative gastrectomy, surgical bypass, endoscopic intervention, palliative chemotherapy, or radiotherapy (6,7,9-12). Of these, palliative external beam radiotherapy (EBRT) has some advantages over others, including greater safety and the fact that it is a non-invasive technique with relatively few restraints concerning the eligibility for treatment (9). Besides, EBRT is effective in mitigating symptoms, and, as it acts directly on neoplastic cells, it has a reasonable probability of delaying tumor progression. Consequently, EBRT may be indicated even for patients with poor clinical performance and various grades of bleeding. Previous studies have shown that EBRT controls tumor bleeding at a rate between 50% and 91% (13). Although 10 fractions of 300 Gy is one of the most common radiotherapy regimes, several other radiotherapy regimes are used in clinical practice (13). Currently, there are doubts about which is the best schedule to palliate local symptoms as well as whether the bleeding response is a good prognostic marker for survival.

Therefore, in this meta-analysis, we evaluated the treatment outcomes of EBRT to palliate bleeding from GC due to local recurrence/progression. Ultimately, we aimed to determine the relationship between bleeding response and the RT schedule.

## METHODS

This systematic review and meta-analysis was carried out according to the Preferred Reporting Items for Systematic Reviews and Meta-analysis (PRISMA) statement and the Meta-analyses Of Observational Studies in Epidemiology (MOOSE) guideline (14). The requirement for approval from the Ethics Committee was waived. Two reviewers performed the research, selected the articles by title and abstract, and then read the full article.

Two investigators conducted a systematic search of PubMed, the Cochrane Central Register of Controlled Trials, and Embase for studies to assess the treatment outcomes of palliative radiotherapy for GC due to local recurrence or progression. We used the following terms “gastric cancer,” “stomach cancer” and “radiotherapy,” “palliative,” “bleeding,” and other synonyms. The lists containing the articles and reviews were checked, and possible related articles were tracked to complement the electronic query. Searches were performed from January 2000 up to March 2019 and were limited to publications in English.

### Study selection

Only studies evaluating the treatment outcomes of GC were included. Studies that reported bleeding response according to the authors’ criteria and retrospective, prospective, non-randomized, and randomized studies were included, whereas case reports were excluded.

### Patients

We included studies of patients with a diagnosis of GC who were previously treated or not treated, who had local recurrence or progression, and who were currently being treated with palliative radiotherapy because of tumor bleeding.

### Intervention

We evaluated the efficacy of palliative radiotherapy. To this end, studies using any fractionation of EBRT to palliate tumor bleeding due to local recurrence or progression of GC were included. Any EBRT technique [2D, 3D, intensity-modulated radiotherapy (IMRT), or volumetric modulated arc therapy (VMAT)] was permitted.

### Outcomes

The following outcomes were evaluated: bleeding response according to the authors’ definition, survival, time without bleeding, and therapeutic effectiveness. Subgroup analysis was performed to evaluate the BED level and RT technique. The studies were stratified by BED level: BED ≥40 Gy 10, BED from 30 to 39 Gy 10, and BED <30 Gy 10. The studies were divided into two groups (2DRT or 3DRT) according to the RT technique. Meta-regression analysis was performed to evaluate the relationship between the BED and bleeding response. The therapeutic effectiveness ratio was considered as the mean of time with no bleeding/the mean of overall survival time x 100. The therapeutic effectiveness ratio was calculated for each study, and the results were stratified by the BED Gy 10 level.

### Clinical data

Patient data, treatment characteristics, and outcomes were retrieved for all included studies. Data on the following characteristics were retrieved: RT technique, RT schedule, time of follow up, bleeding response, and overall survival. With regard to the study characteristics, the design, sample size, inclusion/exclusion criteria, and follow-up time were assessed. Two reviewers gathered all data for all included studies using a standardized data extraction form. A third reviewer was consulted in the event of disagreement.

### Methodological quality assessment

The potential for risk of bias in the studies was evaluated by two authors independently using methodological index for non-randomized studies (MINORS), the index score utilized for methodological evaluation of non-randomized studies. The items were scored 0 points if not reported; 1 point when reported but inadequate; and 2 points when reported and adequate. The maximum MINOR score is 16 points for non-comparative studies. We considered a low risk of bias when a study fulfilled all MINORS criteria and scored >70% on the global scale. We considered a high risk of bias for all other scores. If only abstracts were available, they were automatically considered to be at high risk of bias. A consensus was reached by the two reviewers, and when there was disagreement, a third reviewer’s opinion was the decisive factor.

### Data synthesis and analysis

The proportion rate and 95% confidence interval (CI) of the events for each evaluated outcome were calculated (15). The I^2^ statistic was used to assess statistical heterogeneity, wherein an I^2^ value of <25% was defined as presenting a low level of heterogeneity (16). The meta-analysis was performed using Open Meta-Analyst, a free open software.

Subgroup analyses were performed to determine whether there was a relationship between the BED and bleeding response. A meta-regression analysis was used to evaluate the relationship between the BED and bleeding. The BED was calculated using the following formula:



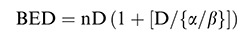



The alpha/beta ratio used for GC was 10, and the estimated survival of responders and non-responders was compared. A *p*-value <0.05 was considered statistically significant in all analyses.

## RESULTS

**Figure 1:**
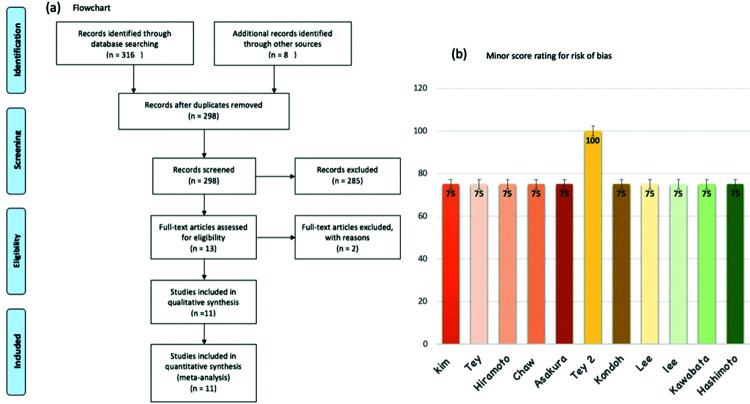
1(a). Flowchart according to PRISMA. 1(b). Minors score rating of each study.

We identified eleven non-comparative retrospective studies, which included 409 patients treated with EBRT to control bleeding due to local recurrence/progression of GC (11,17-26). Figure 1(a). describes the search strategy and the reasons for the exclusion of some studies. Ten of the studies were retrospective (11,17-25), and one was prospective (26); all were published between 2008 and 2019. Gastroscopy was used to confirm gastric bleeding in all patients. The most frequent histology was gastric adenocarcinoma. Regarding the radiation dose, the RT schedule delivering 30 Gy in ten fractions was the most commonly used. The median BED Gy 10 was 39, ranging from 7.2 to 50 Gy 10. 3DRT was used in six studies, 2D RT in three, and RT delivered using mixed techniques in two. Table 1 summarizes the characteristics of the eleven studies. Using the minor score for rating the risk of bias of studies, we stipulated a MINOR score <70% as indicating a high risk of bias. In general, pooling all studies, the mean score was 85% (85%-100%). Only one study achieved an ideal MINOR score of 100%, and this study was also the only prospective study included in our meta-analysis, as presented in Figure 1(b).

### Survival and bleeding response

**Figure 2:**
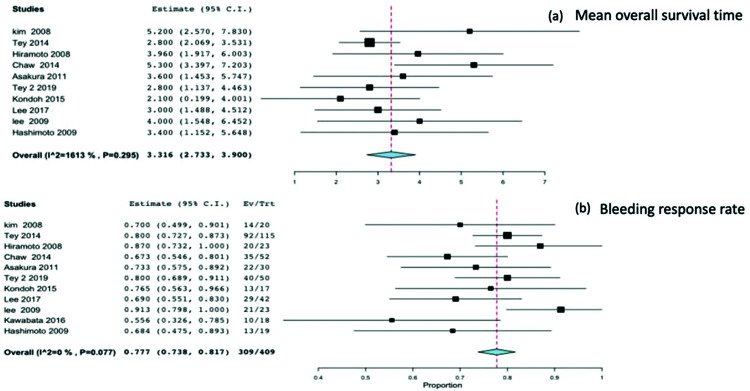
2(a). Mean overall survival time. 2(b). Bleeding response rate.

All studies reported the survival time and bleeding response as outcomes. The eleven studies included a total of 409 patients, and the mean survival reported by all studies was 3.31 months (95%CI 2.73-3.9), with no heterogeneity (*p*=0.295 and I^2^=16%) Figure 2(a). The meta-analysis of all studies reporting the bleeding response rate was 0.77 (95%CI 0.73-0.81), with no heterogeneity (*p*=0.7 and I^2^=0%) Figure 2(b).

### Meta-regression and subgroup analyses for bleeding response and survival

**Figure 3:**
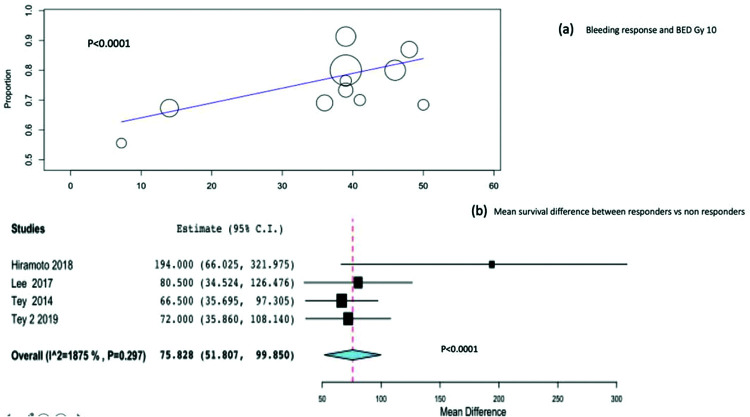
3(a). Meta-regression analysis between bleeding response and BED. 3(b). Subgroup analysis evaluating the mean overall survival difference between responders and non-responders.

We performed meta-regression analysis to identify the relationship between BED Gy 10 and bleeding response. A significant relationship was observed between BED Gy 10 and bleeding response (*p*<0.001), as shown in Figure 3(a).

Four studies, including 230 patients, reported the difference in survival between bleeding responders and non-responders. Combining the four studies, the mean overall survival difference between bleeding responders and non-responders was significant at 75.8 days (95%CI, 51-99; *p*<0.0001) Figure 3(b).

**Figure 4:**
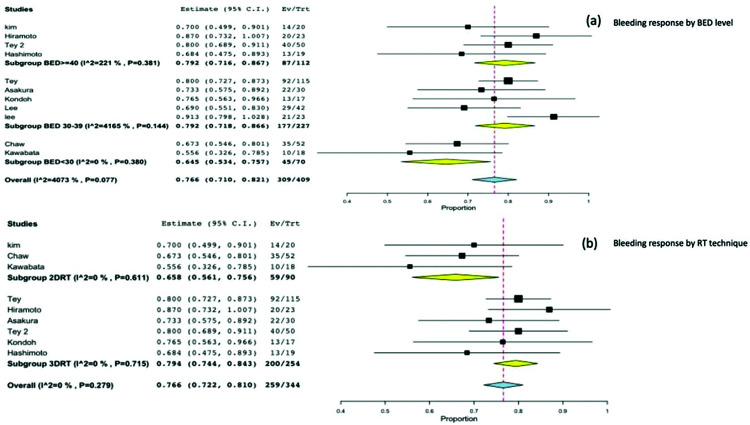
4(a). Subgroup analysis by BED level and bleeding response. 4(b). Subgroup analysis by RT technique and bleeding response.

On stratifying the bleeding response according to the BED Gy 10 level, we found a significantly worse response in the subgroup of studies with a BED <30 Gy 10 (0.64; 95%CI, 0.5-0.7; *p*=0.001), and no significant difference between a BED level of 30-39 Gy 10 (0.79; 95%CI, 0.7-0.8) and a BED level >40 Gy10 (0.79; 95%CI, 0.7-0.8) Figure 4(a).

In the subgroup analysis for the RT technique, we observed a significant difference in bleeding response between 3DRT 0.79 (95%CI, 0.74-0.84) and 2DRT 0.65 (95%CI, 0.56-0.75; *p*=0.021) Figure 4(b).

### Time without bleeding and therapeutic effectiveness ratio

**Figure 5:**
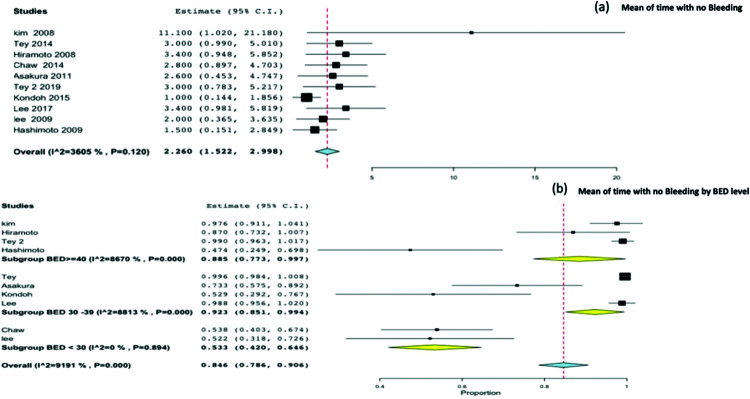
5(a). Mean time with no bleeding. 5(b). Therapeutic effective ratio by BED level.

Ten studies reported the time without bleeding as an outcome. On pooling all studies, the mean time without bleeding was found to be 2.26 months (95%CI, 1.5-2.9) Figure 5(a). The therapeutic effectiveness ratio was 0.84 (95%CI, 0.78-0.90), and by stratifying the therapeutic effectiveness ratio by BED level, we observed a significant difference for BED <30 Gy 10 (*p*<0.001) Figure 5(b).

## DISCUSSION

The present meta-analysis confirms that EBRT is highly effective in stopping bleeding due to local recurrence or progression from GC, with a bleeding response rate of >75% observed by pooling the outcomes of eleven studies.

The high bleeding response rate with palliative radiotherapy is a significant finding for clinical practice, mainly for elderly and fragile patients (Table 1). In recent years, new chemotherapy regimens used to palliate patients with advanced\metastatic GC have improved the survival and quality of life of patients (9). However, many patients with local recurrence from GC are unfit to undergo intensive chemotherapy treatment because of their fragile condition or advanced age. Consequently, palliative RT is an excellent treatment option in this clinical situation.

The quantitative relationship between bleeding response and RT dose is an interesting finding of our study. The meta-regression analysis correlating bleeding response and BED suggests that there is a significant relationship between them and that high BED schedules can result in better bleeding response rates. This information has significant implications in daily clinical practice. Furthermore, this information is novel because a previous systematic review found a lack of dose-response comparing regimens with a BED of >39 Gy 10 *versus* regimens with BED<39 Gy 10 (13). Our data suggest that high BED regimens are capable of producing a better bleeding response and that bleeding responders have improved survival.

We also investigated the ideal BED to palliate gastric tumor bleeding. To this end, we stratified the studies into three levels according to BED Gy 10. Using this approach, the meta-regression analysis indicated a non-significant difference between BED 30-39 Gy 10 and BED ≥40 Gy 10 and a significant difference for BED <30 Gy 10. Notwithstanding, we were unable to establish the ideal cut-off for a better bleeding response; however, our results suggest that a BED >30 Gy 10 should be chosen in clinical practice.

Although the exact mechanism of hemostasis provoked by irradiation is not fully understood, we hypothesized that RT schedules with a higher BED produce extensive damage in the vascular endothelial cells, inducing embolism of vessels, platelet aggregation, and tissue factor release, and subsequently support a better hemostatic response to RT in studies using schedules with higher BEDs.

The hemostatic response to RT was a significant prognostic factor for overall survival. The median survival of responders was significantly longer than that of non-responders (47 *vs* 113.5 days, *p*<0.001). However, the exact relationship between bleeding response and survival remains unclear. The natural explanation is that tumor bleeding could provoke malnutrition, immunosuppression, and dehydration, which could limit adequate cancer treatment (8). Another interpretation would be that in patients with a bleeding response, a high BED has an increased local therapeutic effect on the tumor, which may translate into longer survival. In contrast, we were unable to determine whether the improvement in survival is related to a direct effect of bleeding or if the increase in survival in responders is influenced by other factors, such as chemotherapy, performance status, and the existence of metastasis. However, it is undeniable that patients who achieve a bleeding response have better survival than non-responders, independent of a direct effect or a secondary benefit from the hemostatic response. The therapeutic effectiveness ratio, calculated as the mean time without bleeding after RT and the mean overall survival, reinforces this argument. This index gives us an idea of the importance of the bleeding response over survival. Our data show that, in general, if patients achieves a bleeding response, they have an approximately 85% of chance of no further bleeding at a later stage. Besides, the therapeutic effectiveness ratio shows us that RT schedules with a BED <30 Gy 10 should not be recommended, even for patients with poor clinical performance, because of the lower therapeutic effectiveness compared to that of BED >30 Gy 10 regimens (92% *vs* 53%, *p*=0.001). It is important to note that in the subgroup of studies classified as BED <30 Gy 10 the study conducted by Kawabata et al. (23) used a lower BED (7.2 Gy10), with the possibility of a repeated course in cases in which bleeding did not stop. Although this study has used a completely different treatment schema than that used in the other studies, the statistical difference was maintained even when removing it from analysis, which validated the outcome.

In the literature, mixed RT techniques have been used to palliate local recurrence from GC; among them, the most commonly used techniques are 2DRT and 3DRT. In general, the use of 3DRT allows visualization of the tumor and organs at risk more precisely than that on using 2DRT. Moreover, 3DRT allows us to use multiple fields to deliver a higher radiation dose to the bleeding tumor with better conformality than that on using 2DRT, while also minimizing the higher doses to surrounding organs and tissues. Our analyses detected a significant difference in the rates of bleeding response between the studies using 3DRT and those using 2DRT. Therefore, patients with a high BED schedule would be identified, and the use of 3DRT would improve the chances of success in achieving the bleeding response and, theoretically, better survival as well.

Because there is a dose-response relationship between the BED schedule and the bleeding response, the tumor burden, presence of metastatic disease, and patient performance status are important clinical data for radiation oncologists with regard to the development of personalized radiotherapy schedules. Thus, a short radiotherapy schedule with a high dose per fraction, that is, BED>30 Gy 10, seems adequate for patients who have metastatic disease with a short life expectancy and require urgent symptom control. In contrast, patients with a good performance status, oligometastatic disease, and who are clinically stable could be treated with a longer radiotherapy course, achieving a BED higher than 30 Gy10.

This study provides evidence on the relationship between treatment doses, bleeding response, RT technique, and survival to palliate local recurrence or progression from GC. However, it has some limitations. First, this study is a meta-analysis of retrospective studies that are subject to inherent bias. Second, chemotherapy use, the definition of bleeding response, and treatment techniques were heterogeneous across studies. However, these limitations do not alter any of the outcomes and interpretation derivates of our analysis.

## CONCLUSION

The present meta-analysis confirms that palliative RT is highly effective in controlling gastric bleeding due to local recurrence or progression from GC. Our data suggest a dose-response relationship between bleeding response and BED, and patients with a bleeding response seem to have better survival. Therefore, RT schedules with a high BED should be used in patients with a good performance, oligometastatic disease, and who are clinically stable. Although we did not identify the ideal BED cut-off, our data suggest that BED >30 Gy 10 should be used. Short fractionations with a high dose per fraction, giving a BED >30 Gy 10, should be reserved for patients with a poor clinical performance and in whom bleeding needs to be stopped urgently. Thus, 3DRT should be administered to all patients to maximize the chance of bleeding control and, theoretically, survival.

## AUTHOR CONTRIBUTIONS

Viani GA was the supervisor, responsible for the statistical analyses and manuscript writing. Arruda CV was responsible for the statistical analyses. Hamamura AC, Faustino AC, Danelichen AFB, Matsuura FK and Neves LVF were responsible for the data collection

## Figures and Tables

**Table 1:** Characteristics of studies included in the meta-analysis.

Author, year	N	RT Dose/BED	Age (median)	Metastases	Concurrent chemotherapy	RT technique
Kim et al. (17)	20	35 Gy in 14 fx 41 Gy 10	66	Yes (73%)	Yes (65%)	2DRT
Lee et al. (11)	23	30 Gy in 10 fx 39 Gy 10	69	Yes (96%)	NR	2DRT/3DRT
Hashimoto et al. (18)	19	50 Gy 10	61	NR	Yes (21%)	3DRT
Asakura et al. (19)	30	30 Gy in 10 fx 39 Gy 10	69	Yes (96%)	Yes (40%)	3DRT
Tey et al. (20)	115	30 Gy in 10 fx 39 Gy 10	70	Yes (67.8%)	No	3DRT
Chaw et al. (21)	52	8 Gy in 1 fx (75%) 20 Gy in 5 fx (25%) 14.4 Gy 10	70	Yes (44%)	NR	2DRT
Kondoh et al. (22)	17	30 Gy in 10 fx 39 Gy10	61	Yes (90%)	Yes (33%)	3DRT
Kawabata et al. (23)	18	6 Gy in 3 fx 7.2 Gy 10	69	NR	Yes (11%)	2DRT
Lee et al. (24)	42	39.6 Gy in 20 fx 36 Gy 10	69	Yes (16.7%)	Yes (83%)	2DRT/3DRT
Hiramoto et al. (25)	23	40 Gy in 20 fx 48 Gy 10	69	Yes (91%)	Yes (43%)	3DRT
Tey et al. (26)	50	36 Gy in 12 fx 46 Gy 10	70	Yes (74%)	No	3DRT

2DRT: Conventional radiotherapy, 3DRT: Conformational radiotherapy, BED: Biological effective dose, fx: Fractions.
